# Clinical feasibility of cognitive behavioural therapy for insomnia in a real-world mixed sample at a specialized psychiatric outpatient clinic

**DOI:** 10.1186/s12888-022-04231-4

**Published:** 2022-09-09

**Authors:** Maria Cassel, Kerstin Blom, Jannis Gatzacis, Peter Renblad, Viktor Kaldo, Susanna Jernelöv

**Affiliations:** 1 Centre for Psychiatry Research, Department of Clinical Neuroscience, Karolinska Institutet, & Stockholm Health Care Services, Region Stockholm, Stockholm, Sweden; 2 Stockholm Health Care Services, Region Stockholm, Stockholm, Sweden; 3 Department of Psychology, Faculty of Health and Life Sciences, Linnaeus University, Växjö, Sweden; 4 Division of Psychology, Department of Clinical Neuroscience, Karolinska Institutet, Stockholm, Sweden

**Keywords:** Cognitive behavior therapy, Insomnia, Depression, Anxiety disorder, PTSD, Treatment feasibility

## Abstract

**Background:**

A majority of psychiatric patients suffer from insomnia or insomnia-like problems. In addition to impairing quality of life, sleep problems can worsen psychiatric conditions, such as depression and anxiety, and can make treatment of various psychiatric conditions less successful. Several international guidelines recommend cognitive behavioural therapy for insomnia (CBT-I) as first line treatment. However, patients in psychiatric care are rarely offered this treatment, and there is a lack of studies evaluating the treatment in regular psychiatric settings. In this pilot study, we aimed to determine the clinical feasibility of a group-based CBT-I intervention in an outpatient clinical setting for patients with depression, bipolar disorder, anxiety disorders and PTSD. We also aimed to investigate if symptoms of insomnia, depression and anxiety changed after CBT-I.

**Methods:**

Seventeen patients at an out-patient psychiatric clinic for mixed psychiatric problems of anxiety, affective disorders and PTSD, were enrolled in a six-week long group-based CBT-I intervention. Primary outcomes were pre-defined aspects of treatment feasibility. Secondary outcomes were changes in self-reported symptoms of insomnia severity, depression, and anxiety between pre – and post intervention. Assessment of insomnia severity was also performed 3 months after treatment. Feasibility data is reported descriptively, changes in continuous data from pre- to post-treatment were analysed with dependent t-tests.

**Results:**

All feasibility criteria were met; there were enough patients to sustain at least one group per semester (e.g., minimum 8), 88% of included patients attended the first session, mean of attended sessions was 4.9 of 6, and drop-out rate was 5.9%. Therapists, recruited from clinical staff, found the treatment manual credible, and possible to use at the clinic. Symptoms of insomnia decreased after treatment, as well as symptoms of depression and anxiety.

**Conclusion:**

CBT-I could prove as a clinically feasible treatment option for insomnia in a psychiatric outpatient setting.

**Trial registration:**

Clinicaltrials.gov identifier: NCT05379244. Retrospectively registered 18/05/2022.

## Introduction

Insomnia and insomnia-like sleep problems are widespread, and affect approximately 25% of the population [1]. For patients with psychiatric disorders, these numbers are magnified. Research shows that 45–70% of patients with depression, bipolar disorder, anxiety disorder and PTSD report significant sleep disruption [2, 3].

Sleep has a complex relationship with a variety of psychiatric symptoms. To mention a few, patients with insomnia show more severe levels of depression and anxiety, compared to patients without insomnia [4] and psychiatric patients with depression and comorbid insomnia are less likely to respond to treatment for depression [5]. Insomnia is a risk factor for depression, anxiety, alcohol abuse and psychosis [6] and patients with insomnia, both with and without psychiatric disorders, have an increased risk of suicidal behaviour [7]. Furthermore, psychiatric patients with comorbid insomnia report a higher degree of functional impairment and are unable to perform daily chores to a higher degree than patients without insomnia [3]. Taken together, proper treatment for insomnia is likely to be an important factor in reducing and preventing other psychiatric illnesses and increase everyday functionality.

Traditionally, insomnia has been viewed as secondary to psychiatric diagnoses and was believed to be alleviated as the primary diagnoses were treated. However, there is evidence that residual symptoms of insomnia are common, even after successful treatment of the psychiatric problems [8]. In the scientific literature, residual symptoms of insomnia have been found after successful treatment for PTSD [9] and depression [10, 11] and treating depression does not necessarily alleviate insomnia symptoms [12, 13]. For anxiety, there is some evidence that insomnia symptoms can improve after successful treatment of the anxiety disorder, especially in patients with GAD, but a significant portion of patients do not improve [14, 15]. Hence it seems like insomnia needs targeted treatment.

Cognitive behavioural therapy for insomnia (CBT-I) is an effective treatment for insomnia [16], that has been proven efficacious for patients with psychiatric comorbidities [17], and is recommended as first line intervention in several international guidelines [18–20]. In spite of this, implementation of CBT-I in regular clinical practice is slow and it is estimated that only 1 % of insomnia patients are offered CBT-I [21]. Furthermore, with a few exceptions there is a lack of studies that evaluate the treatment in “real-world settings”, e.g. in regular psychiatric or mental health clinics, and with ordinary staff at the clinics providing the treatments [22]. Our research group has therefore previously performed two studies in regular psychiatric clinics where CBT-I was used for patients with ADHD or bipolar disorder and comorbid sleep problems, with promising results [23, 24]. In these studies, the treatment content was specifically tailored to fit the needs of these specific patient groups and clinics. However, it is not unusual for psychiatric clinics to serve patients with a wide variety of diagnoses. Therefore, it is important to evaluate if the treatment is also feasible and effective when given at clinics with mixed patients, where patients with different psychiatric disorders are included in the same treatment groups.

## Materials and methods

### Aims

Our primary aim is to evaluate if a group intervention based on CBT-I is feasible in a regular psychiatric outpatient clinic for patients with depression, bipolar disorder, anxiety disorder and PTSD. Our second aim is to investigate if symptoms of insomnia, depression and anxiety improve after treatment.

### Design

This is an uncontrolled treatment feasibility study with 17 participants. Feasibility measures were collected throughout the recruitment and treatment process. Symptoms of insomnia, depression and anxiety were assessed pre – and post treatment. Insomnia severity was also assessed at three-month follow-up.

### Participants and recruitment

The study included 17 adult patients at a psychiatric out-patient clinic in Stockholm, Sweden, serving patients with affective and anxiety disorders and/or PTSD. In addition to these diagnoses, a notable minority of patients at the clinic have comorbid conditions like ADHD, autism, substance use disorder or personality disorder.

**Fig. 1:**
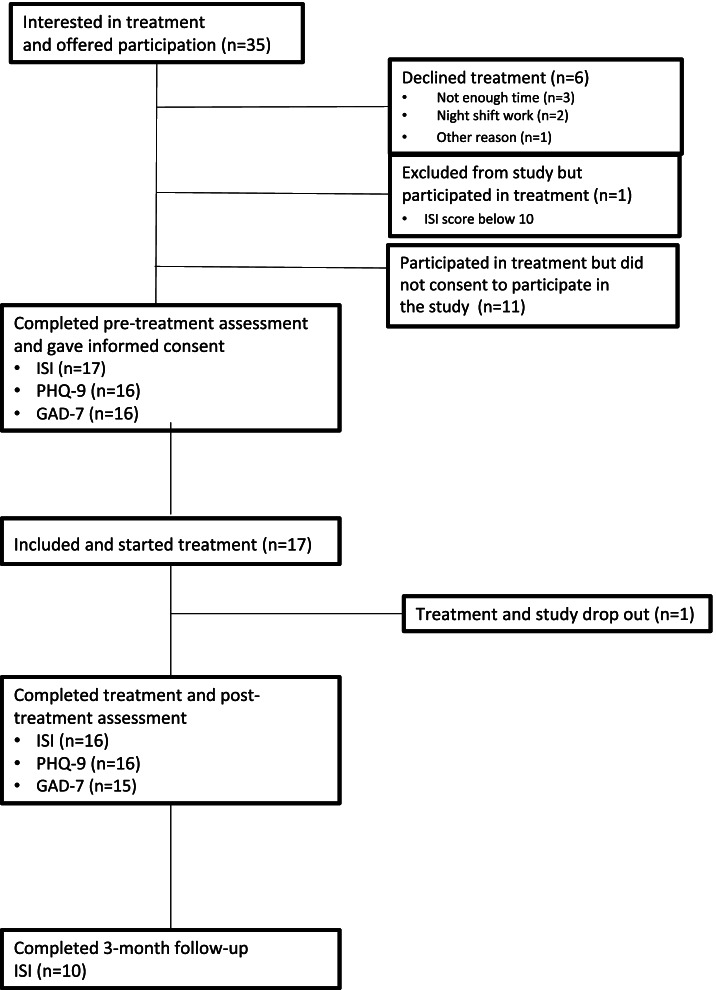
Participant flow through study

Inclusion criteria for the study were self-reported difficulties initiating or maintaining sleep, Insomnia Severity Index (ISI) score > 10, indicating clinical insomnia [25], sufficient knowledge in Swedish and no practical barriers to participate in the group treatment. Exclusion criteria was night shift work and ongoing alcohol or drug abuse that require treatment at a specialised unit. Information about the study was posted in the waiting room and in psychoeducational group meetings, along with registration forms. Patients could also be referred by their doctor, psychologist, or nurse. Patients interested in participating received written information about the treatment and were contacted by telephone for a short interview to assess inclusion- and exclusion criteria. All patients who were interested in the treatment and fulfilled inclusion criteria received treatment. Those not consenting to participate in the study but wanting to participate in the treatment still received the treatment within regular care and some feasibility measures could still be used for those patients, but self-report data is not available. For participant flow, please see Fig. 1.

Patient feasibility measures were collected throughout recruitment, treatment, and follow-up. Therapist feasibility measures were collected post treatment.

### Treatment

The treatment consisted of six group sessions (four to eight participants; first two sessions 120 minutes, the following 90 minutes) led by two therapists. It was based on well-established CBT techniques for insomnia, including sleep-restriction, stimulus control, sleep hygiene, cognitive techniques and relaxation [26]. The content was adapted to the psychiatric patient group in several ways. First, scheduled sleep with the option of sleep compression was introduced as an alternative to sleep restriction. Although the evidence base for sleep compression is far behind that of sleep restriction, in later years it has been suggested as a more gentle method, with fewer adverse events, that can be considered when treatment has to be modified according to patient needs [27]. To enhance treatment adherence, patients were allowed to choose freely between sleep restriction, and scheduled sleep with the option of sleep compression. Patients with bipolar disorder were instructed to choose scheduled sleep in order to avoid sleep deprivation that might trigger hypomania or mania [28]. For patients who chose sleep restriction, a sleep window was set to average total sleep time based on 1 week of sleep diary data. Patients who chose scheduled sleep were instructed to set their sleep window based on estimated sleep need. If they did not know, they were advised to choose a sleep window of 7–7,5 hours since this is suggested to be optimal sleep duration for adults [29]. Time of awakening was set individually based on the patient’s daily schedule or, if possible, patient preference. Sleep schedule was changed weekly based on sleep diary data, adding 15 min by going to bed earlier if sleep efficiency (SE) was > 85; held constant if 80 < SE > 85; and shortened by 15 min by going to bed later if SE < 80. Patients with a mean total sleep time of less than 5 h were instructed to choose sleep compression and use it together with stimulus control if unable to sleep. Second, instructions for stimulus control were slightly adjusted. Instead of giving the instruction of going to bed only when sleepy, patients were instructed to follow their sleep window. If not being able to fall asleep or if they woke up during the night, they were instructed to leave the bed for 15–20 minutes and then go back and try to fall asleep again. This was to minimize the risk of patients engaging in arousing activities that could interfere with sleep and because some patients have a high level of arousal that makes it difficult for them to notice sleepiness. Third, interventions aiming to stabilize circadian rhythm were added, including systematic use of light and darkness, i.e. light exposure in the morning (e.g. draw the blinds and turn on the lights when waking up, take a morning walk, and dim lights at night for evening chronotypes and the opposite for morning chronotypes), as well as regular meals and wake-up and wind-down routines [30, 31]. Fourth, psychoeducation included information on sleep and psychiatric disorders. Detailed information of treatment content can be found in Table 1.

**Table 1:** Treatment content

Session	Content	Homework
1	Mindfulness exercise. Psychoeducation about sleep and psychiatric disorders, relaxation, sleep hygiene. Goal setting.	Read summary of session. Keep sleep diary. Chose 1–2 sleep hygiene strategies and use every day. Do one relaxation exercise a day
2	Mindfulness exercise. Sleep restriction, scheduled sleep with option of sleep compression, stimulus control. Strategies to wake up in the morning and wind down at night	Read summary of session. Keep sleep diary. Stick to sleep window. Use stimulus control. Do one relaxation exercise a day. Optional to use other strategies, but participants are encouraged to try some additional strategies for at least one week.
3	Mindfulness exercise. How light, meals, and activities affects the circadian rhythm. Introduction to worry time. Adjust sleep window.	Read summary of session. Keep sleep diary. Stick to sleep window. Use stimulus control. Do one relaxation exercise a day. Optional to use other strategies.
4	Mindfulness exercise. Evaluation of treatment goals, exploring pros and cons with using new sleep related behaviour. Adjust sleep window.	Read summary of session. Keep sleep diary. Stick to sleep window. Use stimulus control. Do one relaxation exercise a day. Optional to use other strategies.
5	Mindfulness exercise. Summary of treatment. Addressing questions and ambiguities. Adjust sleep window.	Read summary of session. Keep sleep diary. Stick to sleep window. Use stimulus control. Optional to use other strategies.
6	Mindfulness exercise. Summary of treatment content. Evaluation of treatment goals. Creating individual sleep plan. Relapse prevention	Follow sleep plan

To facilitate therapist treatment adherence, all sessions had a power-point presentation with a manuscript for each slide. At the end of each session, patients received a booklet with a summary of session content and all worksheets for the session. The booklet was also sent home to patients who did not attend the session.

### Therapists

A total of four therapists from the regular staff acted as group leaders. Two of them were licensed psychologists, one a psychologist in training and one a psychiatrist, all with at least 18 months of training in CBT and with additional training in CBT-I. The therapists had access to supervision on demand by last author SJ.

### Outcome measures

#### Treatment feasibility

Feasibility was measured in several ways related to uptake, attendance, and acceptability. The feasibility criteria were formulated in collaboration with staff at the clinic and reflect their view on what criteria a treatment should meet to be considered feasible in the real-world clinical setting.

*Patient related feasibility criteria*, collected throughout recruitment and treatment process

- Number of patients interested in the treatment after given information is sufficient to start at least one group per semester (i.e., around eight).
- At least 50% of included patients attend the first session
- Patients attend on average at least three out of six sessions
- Treatment drop-out, defined as attending less than three sessions in total, is below 50%

All four patient-related feasibility measures were registered for all patients, also patients who did not participate in the study.

*Therapist related feasibility measures*, collected after all treatment groups included in the study had ended treatment.

- Therapists find the treatment manual credible, assessed by semi-structured interviews
- Therapists want to continue using manual after end of study, assessed by semi-structured interviews

#### Symptom of insomnia

Symptoms of insomnia were measured using the Insomnia Severity Index (ISI) at pre-, post-, and three-month follow-up. The ISI is a 7-item self-report scale that assesses symptoms of insomnia. It has adequate psychometric properties and is sensitive to change [32]. Each item is scored on a 0–4 scale, total score ranging from 0 to 28, higher score representing a higher degree of symptoms. A cut-off score of 10 indicates probable insomnia in a community sample [25]. Levels of insomnia severity were defined as: 0–7 points: no clinically significant insomnia, 8–14 points: sub-threshold insomnia, 15–21 points moderate insomnia, 22–28 points: severe insomnia [25].

#### Symptoms of depression

Patient’s health questionnaire-9 (PHQ-9) was administered at pre – and post-treatment. The PHQ-9 is a 9-item self-report scale with good psychometric properties, which assesses each of the nine DSM-5 diagnostic criteria of depression on a 0–3 scale, total score ranging from 0 to 27. A higher score represents a higher level of depression symptoms. Depression severity was defined according to Kroenke as minimal (0–4), mild (5–9), moderate (10–14), moderately severe (15–19) or severe depression (20–27) [33].

#### Symptoms of anxiety

Generalised anxiety disorder scale 7 (GAD-7) was administered at pre – and post-treatment. The GAD-7 is a reliable and valid 7-item self-report scale used as a tool for screening for GAD with 10 points recommended as cut-off score for likely generalized anxiety disorder [34]. In an outpatient setting for patients with anxiety and affective disorders, high scores mirror general distress and negative affect in patients [35]. Each item is scored on a 0–3 scale, total score ranging from 0 to 21. A higher score represents a higher level of symptoms. Levels of anxiety were defined as mild (5–9), moderate (10–14) and severe (15–21) [34].

### Power considerations

Based on previous research on CBT-I for patients with psychiatric disorders, within-group effect size on the secondary outcome measure ISI, was estimated to Cohen’s d = 1.0 [36, 37]. To achieve a power of 80% with an alpha-level of 0.05, 16 participants were needed. Since attrition was expected, we aimed for 20 participants. This number of patients was also deemed to be enough to evaluate the primary outcomes related to feasibility.

### Data handling and statistical analysis

Feasibility data is reported descriptively.

Statistical analysis was made using Jamovi, version 1.6.23 [Computer Software]. Retrieved from https://www.jamovi.org.

Data was screened for normality using the Shapiro Wilks test, and no data violated the assumption of normality.

One participant was missing one individual item from one assessment point for the GAD-7. This item was imputed using the corrected item mean substitution [38].

Changes in continuous data from pre- to post-treatment was analysed with dependent t-test, in a per protocol analysis, including those patients who had completed at least three out of six sessions, since this dose could render an effect on insomnia severity. The within-group effect size was calculated on observed data and is reported as Cohen’s *d* with 95% confidence intervals [39]. Pooled SD was used as denominator: (SDpre+SDpost)/2. Sensitivity analyses were performed using dependent t-tests with last observation carried forward (LOCF) for participants with pre- but not post-data.

Differences in severity levels for insomnia, depression and anxiety were analysed with Wilcoxon signed ranks test on observed data at pre- and post-treatment.

Due to high attrition at three-month follow-up, no statistical tests were performed on follow-up data, but results are presented numerically and graphically.

## Results

### Data attrition

One participant did not fill out pre-assessments of PHQ-9 and GAD-7, one participant did not complete any of the post-assessment forms and one participant did not fill out post-assessment of GAD-7. Seven participants (41%) were missing from the three-month follow-up.

### Participants

As can be seen in Table 2, a majority of participants were female. All participants had experienced sleep problems for more than 3 months, and 93% for at least a year, with an average of 3 years. A majority, 94%, had at least one type of sleep medication prescribed, the most common being antihistamines followed by non-benzodiazepines (z-drugs). All patients had an ISI-score of 11 or higher, indicating clinical insomnia [25].

**Table 2:** Participant demographics, clinical profile, and use of medication pre treatment

Variable	Total *n* = 17
**Age,** M (range)	47 (20–87)
**Sex**	
Female, N (%)	11 (65%)
**Educational level**	
Primary school	1 (6%)
Secondary school	12 (71%)
University	4 (24%)
**Occupation**	
Employed/student	12 (71%)
Unemployed	3 (18%)
Retired	3 (18%)
**Sick leave** (*n* = 15)^a^	
100%	2 (12%)
75%	2 (12%)
50%	2 (12%)
25%	3 (18%)
Not on sick leave	8 (47%)
**Psychiatric diagnosis currently treated at the unit**	
PTSD	5 (29%)
Depression or dystyhimia	4 (24%)
Bipolar disorder 2	3 (18%)
Bipolar disorder 1	2 (12%)
Anxiety disorder	2 (12%)
Other	1 (6%)
**Comorbid psychiatric disorders**^b^	
Alcohol and/or substance abuse/addiction	4 (24%)
ADHD	3 (18%)
Depression	2 (12%)
Anxiety disorders	1 (6%)
Other	1 (6%)
**Duration of sleep problems**	
Mean years (range)	3 (0.4–10 years)
0,4-0,9	3 (18%)
1–2	8 (47%)
2,1–5	3 (18%)
5,1–10	3 (18%)
**Pre-treatment Insomnia Severity**	
Mean ISI score (SD)	19.5 (4.7)
Clinical insomnia, severe (22–28 points)	5 (29%)
Clinical insomnia, moderate severity (15–21 points)	10 (59%)
Sub-threshold insomnia (8–14 points)	2 (12%)
No clinically significant insomnia (0–7)	0 (0%)
**Sleep medications n (%)**	
None	1 (6%)
One type of drug (e.g. antihistamine or z-drug)	9 (53%)
Two types of drugs (e.g. antihistamine *and* z-drug)	7 (41%)
**Type of sleep medication**^c^ **n (%)**	
Antihistamines	9 (53%)
Z-drugs	8 (47%)
Benzodiazepines	3 (18%)
Melatonin	1 (6%)
Propiomazin	1 (6%)
**Duration of sleep medication use n (%)**	
Mean years (range)	2.9 (0.4–10)
0,4-0,9	2 (12%)
1–2	8 (47%)
2,1–5	3 (18%)
5,1–10	3 (18%)
**Other medications** ^c^ **n (%)**	
Antidepressant (SSRI, SNRI, NaSSA, SNRI, NDRI)	13 (76%)
Lithium	4 (24%)
Anticonvulsant (e.g. valproat)	3 (18%)
Stimulants (e.g. methylfenidate)	3 (18%)
Antipsychotics (e.g. qeutiapin)	3 (18%)
Opioid substitution drug	1 (6%)
Benzodiazepines	1 (6%)

^a^pensioners are not included since they can not be on sick leave

^b^particapants can have one or several of these diagnoses

^c^particapants can use one or several of these drugs

The most common primary psychiatric diagnosis was PTSD followed by depression, with 29 and 24% of participants respectively. The second most common was bipolar disorder type II, with 18%. Forty-one percent of the patients had at least one comorbidity, the most common being substance abuse.

### Treatment feasibility

As can be seen in Table 3, all feasibility criteria used in the study were met.

**Table 3:** Feasibility criteria and outcome

	Outcome
**Patient related feasibility criteria**	
Number of patients interested in the treatment after given information, is sufficient to start at least one group per semester (i.e. around eight).	Yes. M = 11.6 interested patients per semester
At least 50% of included patients attend the first session	Yes. 88% attended first session
Patients attend on average at least three out of six sessions	Yes. M = 4.9 sessions out of 6
Treatment drop-out, defined as attending less than three sessions in total, is below 50%	Yes. Drop-out rate 5.9%
**Therapist related feasibility criteria**	
Therapists find the treatment manual credible	Yes. All four therapists found the manual credible
Therapists want to continue using manual after end of study	Yes. All four therapists wanted to use the manual after the end of study

In total, 29 participants took part in the treatment. In the whole group, 76% of these patients attended the first session, mean session attendance was four sessions out of six and drop-out rate was 21%, thus these feasibility criteria were well met when looking at the whole group as well.

### Symptoms of insomnia, depression, and anxiety

#### Symptoms of insomnia

As can be seen in Table 4, symptoms of insomnia decreased after treatment. A completer analysis with dependent t-test showed statistically significant reductions of the ISI score from pre-to post treatment (t(15) = 6.4, *p* < .001, *d* = 1.6).

**Table 4:** Means, standard deviations and effects sizes for symptom measures, observed values

Measure	Pre-treatment		Post- treatment		3-month follow-up		Within group effect size d [95%CI]
	N	M (sd)	N	M (sd)	N	M (sd)	Pre-post
ISI	17	19.5 (4.7)	16	9.9 (5.5)	10	7.8 (4.7)	1.6^***^ [0.8–2.3]
PHQ-9	16	13.1 (6.0)	16	7.3 (6.1)	–	–	0.9^**^ [0.3–1.5]
GAD-7	16	8.6 (4.4)	15	7.0 (4.9)	–	–	0.4 [−0.1–1.0]

^*^p < .05

^**^p < .01

^***^p < .001

As a sensitivity analysis, an intent-to treat analysis with dependent t-test was carried out for all participants that filled out pre-assessment, using LOCF for one participant for the ISI and one for GAD. Results from this sensitivity analysis did not differ from the main analysis.

**Fig. 2:**
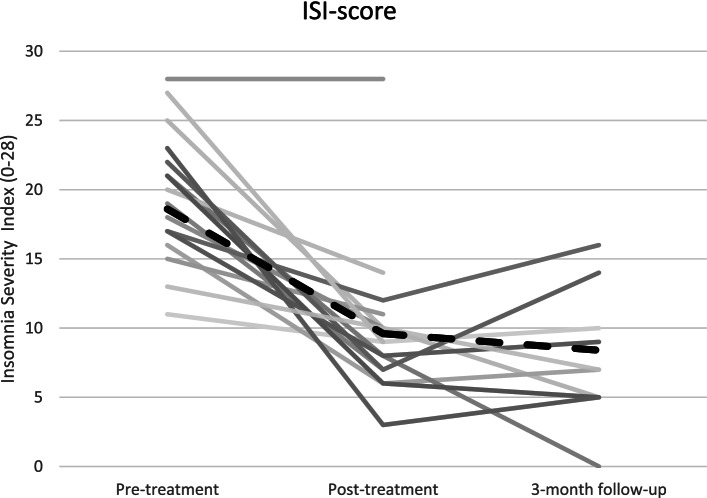
Individual scores on Insomnia Severity Index, observed data, at the three assessment points. Dotted black line represents mean ISI-score

Figure 2 shows individual ISI-score at pre-, post- and follow-up-assessment. At post-assessment 10 out of 17 participants (59%) were responders, i.e., had a decrease in ISI-score with 8 points or more. At three-month follow-up, 7 out of the 10 participants (70%) that filled out assessments were responders, ie., had at least 8 points decrease on the ISI compared to pre-treatment assessment.

##### Proportion of patients with different levels of insomnia severity (ISI)

**Fig. 3:**
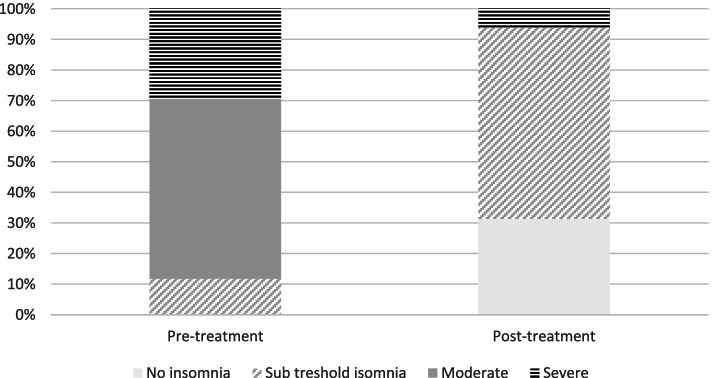
Proportion of patients with different levels of insomnia severity before and after the intervention

Figure 3 shows proportion of patients based on level of insomnia severity pre – and post treatment. As can be seen, patients moved from higher severity level before treatment, to a lower severity level after treatment. Please note that no patient was in the moderate range at post-treatment. Results from Wilcoxon signed ranks tests showed statistically significant changes in distribution of levels of insomnia between pre – and post treatment (W = 91, *p* = .001).

#### Symptoms of depression

As can be seen in Table 4, symptoms of depression decreased after treatment. A completer analysis with dependent t-test showed statistically significant reductions of the PHQ-9- score from pre-to post treatment (t(15) = 3.59, *p* = 0.003, *d* = 0,90).

**Fig. 4:**
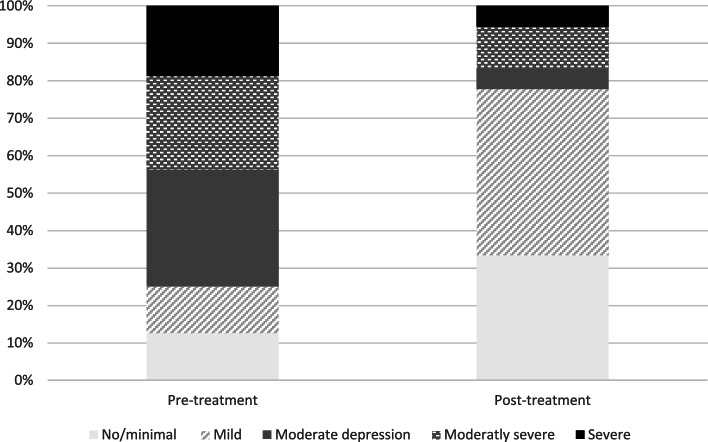
Proportions of patients with different levels of depression before and after the intervention

Figure 4 shows proportion of patients based on level of depression severity pre – and post treatment. As can be seen, patients moved from higher severity level before treatment, to a lower severity level after treatment. Results from Wilcoxon signed ranks tests showed statistically significant changes in distribution of levels of depression between pre – and post treatment (W = 5 0, *p* = .024).

#### Symptoms of anxiety

As can be seen in Table 4, symptoms of anxiety decreased after treatment. A completer analysis with dependent t-test did not show statistically significant reductions of the GAD-7 score from pre-to post treatment (t(14) = 1,54, *p* = 0.146, *d* = 0,4).

**Fig. 5:**
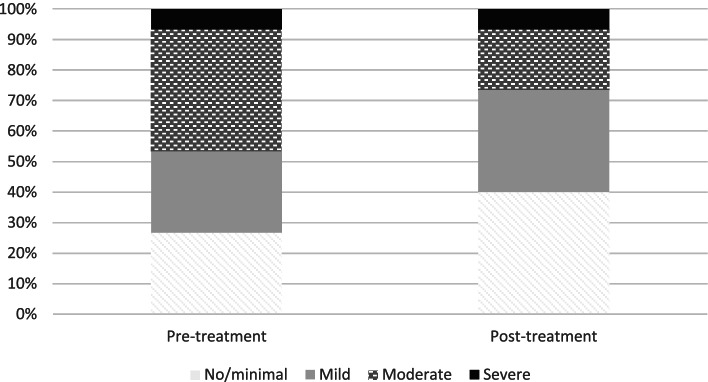
Proportions of patients with different levels of anxiety before and after the intervention

Figure 5 shows proportion of patients based on level of anxiety severity pre – and post treatment. Patients moved from a higher severity level before treatment, to a slightly lower severity level after treatment, but Wilcoxon signed ranks tests showed no statistically significant changes in distribution in different levels of anxiety between pre – and post treatment (W = 18.5, *p* = 0.11).

## Discussion

The primary aim of this study was to evaluate the feasibility of a group intervention to treat sleep problems based on CBT-I, in a real-world setting at an outpatient psychiatric clinic for patients with depression, bipolar disorder, anxiety disorder or PTSD, where patients with different psychiatric disorders were included in the treatment groups. Our second aim was to investigate changes in symptoms of insomnia, depression and anxiety following the intervention.

Our result show that the treatment was feasible in this real-world psychiatric clinic. Patient interest in treatment and session attendance was high, and dropout rates were low. Therapists, that were all regular clinical staff, found the treatment manual credible and useful at the clinic. Symptoms of insomnia and depression improved after treatment and there was a tendency towards a reduction in symptoms of anxiety, although the change in anxiety symptoms was not statistically significant.

This study indicates that CBT-I is a viable treatment option to treat difficulties initiating and/or maintaining sleep for patients with complex psychiatric symptoms, and shows that it is possible to deliver the treatment in a real-world setting of routine psychiatric care. This is especially encouraging since the load of psychiatric symptoms in this group of patients might make it difficult to work with a demanding treatment like CBT-I. In addition, it can be challenging to introduce a novel treatment in an existing clinical practice, with high workload, established treatment routines, and a selection of patients that primarily sought help for other conditions than insomnia. With that in mind, several factors were considered both in terms of treatment content and study design.

Starting with the treatment content, sleep restriction therapy is a common intervention in most CBT-I protocols. While it is an efficient intervention [40] it is also associated with high numbers of adverse events like extreme sleepiness, headache, problems with concentration during the day and depressed mood. These adverse events, together with practical problems like difficulty staying awake until bedtime, and the fact that it can be hard to know what to do during all the extra waking hours, can make patient adherence to sleep restriction therapy difficult [41]. For patients already struggling with symptoms like depression and anxiety this intervention can be even more challenging and might lead to treatment drop-out or lack of treatment compliance. Sleep compression, where time in bed is gradually reduced to match actual sleep [42], is as far as we know now not associated with those side effects [43]. To facilitate participation in the treatment and prevent dropouts due to adverse events, we let the patients choose whether to use sleep restriction or scheduled sleep with the option of sleep compression. By adding this opportunity, patients who perceived a short sleep window to be too demanding could choose a less challenging treatment option. The possibility to choose a preferred intervention has previously been associated with lower drop-out rates and stronger therapeutic relationship [44]. Unfortunately, we do not have data on which of these interventions patients chose. Another challenge with CBT-treatments is to plan and perform homework assignments. Considering the diversity among patients in this study, both in terms of diagnoses and level of functioning, therapists were instructed to allocate time at the end of session to help patients plan, and sometimes adapt, homework assignments. Assignments that match the patients level of functioning together with a clear structure for assignments has been suggested to promote homework compliance [45].

As mentioned in the introduction, access to CBT-I is limited in regular clinical practice, although it has been researched for half a century and is recommended as first line treatment for insomnia in several international guidelines (e.g., [18–20]). In Sweden it is estimated that only approximately 2000–3000 patients in total have been treated with CBT-I [46], although prevalence of insomnia and insomnia-like sleep problems is estimated to 25% [1], indicating that around 2 million Swedish adults could benefit from treatment for insomnia, a proportion of whom are treated in psychiatric care. However, this is not unique to CBT-I. To disseminate and implement new treatments in health care has proven difficult. In 2000, Balas and Boren stated that it takes an average of 17 years for research findings to reach clinical practice [47] an estimate that largely still holds true [48].

It has been suggested that one way to facilitate implementation of treatments into clinical reality, is to reflect on factors that can support or hinder implementation throughout the research process [49]. Another key issue is that scientists must adapt interventions to fit into real-world conditions, while keeping core components from the treatment [50]. With that in mind, the manual and design of this study were developed in close collaboration with clinicians and executives at the psychiatric clinic where the study took place. This was to ensure that they found the treatment valid for their patients and thereby meaningful to allocate resources to, and to plan the practical arrangements in a way that made the treatment and study possible to fit into the clinical routine. Another important topic when it comes to implementation is to make sure that treatment is delivered as intended [51]. In this study, this was ensured in several ways regarding therapist training, materials, and supervision, as described in the methods section.

Only one participant dropped out of treatment, giving a drop-out rate of 6 % which is a very low number for CBT-treatments. However, this number might be too optimistic since patients were offered treatment regardless of their participation in the study. In total, 29 patients took part in the treatment, but only 17 of them participated in the study. From all 29 patients who started treatment, a total of six patients dropped out (one being a study participant). This gives a drop-out rate of 20%, a number more in line with the 26% reported from other studies of CBT-treatments [52–54].

Patients reported improvements in insomnia symptoms after the treatment. This indicates that CBT-I can be a treatment option that reduces symptoms of insomnia for psychiatric patients. Our findings are in line with previous studies on CBT-I for patients with psychiatric diagnoses [23, 24, 36, 55]. We also found a statistically significant improvement in depressive symptoms, with a moderate effect size. This result adds to the increasing number of studies where treatment with CBT-I has been shown to have a positive effect on symptoms of depression as well as symptoms of insomnia [12, 56–59]. We also saw a decrease in anxiety symptoms after treatment, with a small effect size, similar to earlier studies [60]. This decrease was not statistically significant, likely due to the small sample size in the current study.

## Strengths and limitations

An important limitation to this study is the lack of control group and the fact that not all patients who received the treatment consented to participate in the study. The lack of control group means that it is not possible to conclude that the observed decrease in symptoms of insomnia and co-morbid symptoms is a result of the treatment. Since the patient group is varied with several different diagnoses it is not possible to draw any conclusions regarding which of the treatment components might have contributed to the observed effects or if some components worked better for a specific patient group. Another important limitation is that data on medication use was not collected after treatment. This means that it cannot be excluded that the observed changes in sleep and comorbid symptoms are due to changes in medication or effects of an effective pharmacological regime. In addition, the follow-up period is short and only ISI was measured at three-month follow-up, with high attrition; no objective sleep measures were collected, and it is therefore not possible to analyse if reductions in ISI is reflecting actual changes in sleep parameters such as sleep onset latency or wake after sleep onset.

The study also has several strengths: Most importantly, the study has very high ecological validity. It was conducted in a real-world regular psychiatric clinic, with generous inclusion criteria to allow for a majority of interested patients to be included. Group leaders were regular staff at the clinic, who participated in the study alongside their ordinary tasks. This means that the treatment has potential to be accepted and implemented also at other psychiatric clinics. Other strengths are the low drop-out rate and low data attrition at post-treatment assessment. To further confirm and elaborate these findings, a randomized controlled trial is currently being conducted. Further studies are also needed to investigate treatment processes and mediators of treatment effects.

## Conclusion

This study indicates that CBT-I could be a feasible and effective treatment option for psychiatric patients in routine specialized psychiatric care. Patient interest in the treatment was high, as was session attendance, and treatment drop-out was low. Therapists found the treatment manual credible and easy to work with. Symptoms of insomnia improved after treatment, as did symptoms of depression. An important contribution of this study to the scientific literature on CBT-I is that it shows that it is possible for clinicians to deliver CBT-I to groups of patients with sleep problems comorbid with mixed psychiatric conditions, as a part of real-world, everyday clinical work. Although more research is needed before firm conclusions can be drawn, the study suggests that CBT-I, also when given in this context, may be able to decrease symptoms of insomnia and comorbid psychiatric symptoms.

## Data Availability

The datasets generated for this article are not readily available because patient data sharing is restricted by Swedish law and GDPR. Requests to access the datasets should be directed to maria.cassel@ki.se.

## Abbreviations

CBT: Cognitive behavioural therapy
CBT-I: Cognitive behavioural therapy for insomnia
ISI: Insomnia Severity Index
PHQ-9: Patient’s health questionnaire-9
GAD-7: Generalised Anxiety disorder scale-7
PTSD: Post traumatic stress disorder
ADHD: Attention Deficit Hyperactivity Disorder
ASD: Autism Spectrum Disorder
